# COVID-19 lung CT image segmentation using deep learning methods: U-Net versus SegNet

**DOI:** 10.1186/s12880-020-00529-5

**Published:** 2021-02-09

**Authors:** Adnan Saood, Iyad Hatem

**Affiliations:** Mechatronics Program for the Distinguished, Tishreen University, Distinction and Creativity Agency, Latakia, Syria

**Keywords:** COVID-19, Pneumonia, SegNet, U-NET, Computerized tomography, Semantic segmentation

## Abstract

**Background:**

Currently, there is an urgent need for efficient tools to assess the diagnosis of COVID-19 patients. In this paper, we present feasible solutions for detecting and labeling infected tissues on CT lung images of such patients. Two structurally-different deep learning techniques, SegNet and U-NET, are investigated for semantically segmenting infected tissue regions in CT lung images.

**Methods:**

We propose to use two known deep learning networks, SegNet and U-NET, for image tissue classification. SegNet is characterized as a scene segmentation network and U-NET as a medical segmentation tool. Both networks were exploited as binary segmentors to discriminate between infected and healthy lung tissue, also as multi-class segmentors to learn the infection type on the lung. Each network is trained using seventy-two data images, validated on ten images, and tested against the left eighteen images. Several statistical scores are calculated for the results and tabulated accordingly.

**Results:**

The results show the superior ability of SegNet in classifying infected/non-infected tissues compared to the other methods (with 0.95 mean accuracy), while the U-NET shows better results as a multi-class segmentor (with 0.91 mean accuracy).

**Conclusion:**

Semantically segmenting CT scan images of COVID-19 patients is a crucial goal because it would not only assist in disease diagnosis, also help in quantifying the severity of the illness, and hence, prioritize the population treatment accordingly. We propose computer-based techniques that prove to be reliable as detectors for infected tissue in lung CT scans. The availability of such a method in today’s pandemic would help automate, prioritize, fasten, and broaden the treatment of COVID-19 patients globally.

## Background

COVID-19 is a widespread disease causing thousands of deaths daily. Early diagnosis of this disease proved to be one of the most effective methods for infection tree pruning [1]. The large number of COVID-19 patients is rendering health care systems in many countries overwhelmed. Hence, a trusted automated technique for identifying and quantifying the infected lung regions would be quite advantageous.

Radiologists have identified three types of irregularities related to COVID-19 in Computed Tomography (CT) lung images: (1) Ground Glass Opacification (GGO), (2) Consolidation, and (3) pleural effusion [2, 3]. Developing a tool for semantically segmenting medical lung images of COVID-19 patients would contribute and assess in quantifying those three irregularities. It would help the front-liners of the pandemic to better manage the situation of overloaded hospitals.

Deep learning (DL) has become a conventional method for constructing networks capable of successfully modeling higher-order systems to achieve human-like performance. Tumors have been direct targets for DL-assisted segmentation of medical images. In [4], a lung cancer screening tool was implemented using DL structures aiming to lower the false positive rate in lung cancer screening with low-dose CT scans. Also, in [5], researchers attempted to segment brain tumors from MRI images with a hybrid network of U-NET and SegNet, reaching an accuracy of 0.99. Breast tumor was also a target for segmentation in [6] using Generative Adversarial Networks (GANs) and Convolutional Neural Networks (CNNs) resulting in a mean accuracy of 0.90. Body parts were subject to segmentation also; researchers attempted to segment: kidneys in [7], Lungs in [8, 9], liver in [10], brain tissue in [11] and [12], temporal bones in [13], and arterial walls in [14].

Until today, many research projects have been conducted for COVID-19 detection using DL analysis of medical images such as X-Ray and CT scans and revealed significant results. However, semantically segmenting those images has been less appealing.

Many DL structures were considered by researchers to detect COVID-19 patients using medical images. A recent study designed a binary classifier (COVID-19, No information) and a multi classifier (COVID-19 , No Information, Pneumonia) using a CNN with X-Ray images as an input, reaching 0.98 for binary classes and 0.87 for a multi-class classifier [15]. Another study employed Xception and ResNet50V2 networks for COVID-19 detection from CT scans, resulting in an accuracy of 0.99 for the target class [16]. References [17–21] used various DL systems with medical images and obtained results with accuracy values ranging from 0.83 to 0.98.

Few attempts for semantically segmenting medical images of COVID-19 patients were published recently. Implementing such a tool would be a key component in a system that prioritizes patients by severity. It would identify an infection and output its key spatial features such as size, distribution, and shape parameters. Bounding-box segmentors, by definition, fail to deliver such parameters. Thus, we expect it to have poor performance in illness severity assessment. Study [22] employed a deep CNN as a binary segmentor and compared it to other structures (FCN, U-NET, VNET, U-NET++). The authors reached a Sorensen-Dice of 0.73, a sensitivity of 0.75, and a precision of 0.73. Another usage of DL as a binary segmentation tool was presented in [23]. The study reached a Dice of 0.78, an accuracy of 0.86, and a sensitivity of 0.94. Reference [24] implemented a Fully Convolutional Network (FCN) and a U-NET as binary segmentation tools; their work performed well in terms of precision and accuracy, but lesser in terms of recall and Dice.

Researchers in [25] detailed the design of a novel DNN structures named Inf-Net and Semi-Inf-Net to semantically segment infected regions, and to segment GGO and consolidation. Their work utilized the same data set that this research is using. In binary segmentation, their results reached a Dice of 0.74, a sensetivity of 0.72 and specitivity of 0.96. While in Cosilidation and Opacity segmentation, the average results were 0.54, 0.56 and 0.97, respectively.

## Methods

### The dataset

Images of the dataset used in this work is a collection of the Italian Society of Medical and Interventional Radiology [26]. One hundred one-slice CT scans are provided in a resized $512\times 512$ dimensions. Region labels are already compiled into a NIFTI with proper documentation by the author.

In manual labeling, classes pixel count (total number of pixels in a class) and image pixel count (total number of pixels in images that had an instance of a class) show an extensive disparity in representation; the dominant class is larger in order of ${1}{{\mathrm{e}}}{+3}$ than the least represented class. See Table 1. We note here that the class C_0_ not only represent the portions of the lungs unaffected by pneumonia, but also the lung-enclosing tissue.

**Table 1:** Dataset class sizes

Class	Metrics	
Name	Pixel count	Image pixel count
${\hbox {C}}_0$	${2.4394}{{\mathrm{e}}}{+07}$	${2.6214}{{\mathrm{e}}}{+07}$
${\hbox {C}}_1$	${1.1965}{{\mathrm{e}}}{+06}$	${2.5166}{{\mathrm{e}}}{+07}$
${\hbox {C}}_2$	${5.8921}{{\mathrm{e}}}{+05}$	${2.0447}{{\mathrm{e}}}{+07}$
${\hbox {C}}_3$	$3 {3.4265}{{\mathrm{e}}}{+04}$	${6.5536}{{\mathrm{e}}}{+06}$

Pixel Count denotes the total number of pixels of the class, and Image Pixel Count is the total number of pixels of images that had an instance of the class

**Fig. 1:**
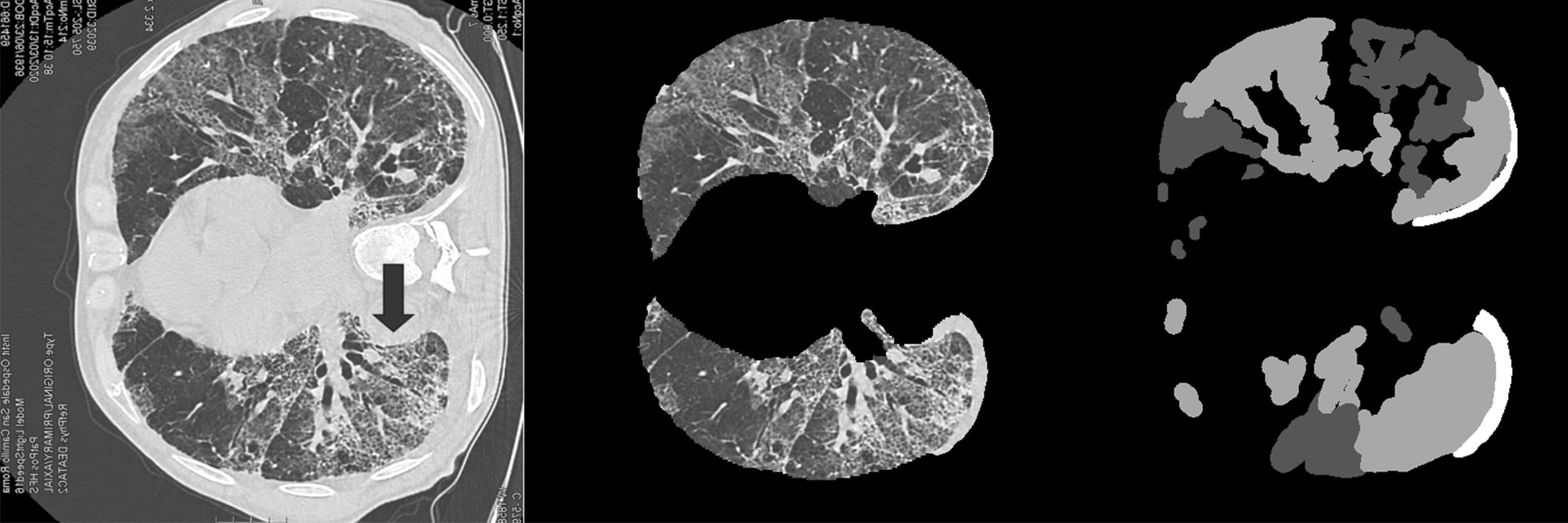
Dataset sample. CT scan (left), masked lungs (middle), and labeled classes (right), where black is class C_0_, dark gray is C_1_, light gray is C_2_, and white is C_3_.

The dataset source website offers image masks to segment the lung regions. These masks were created automatically based on [27]. The automated lung segmentation model can be found in the GitHub repository JoHo/lungmask. Figure 1 illustrates the original, the lung-masked, and the labeled images of one sample.

**Fig. 2:**
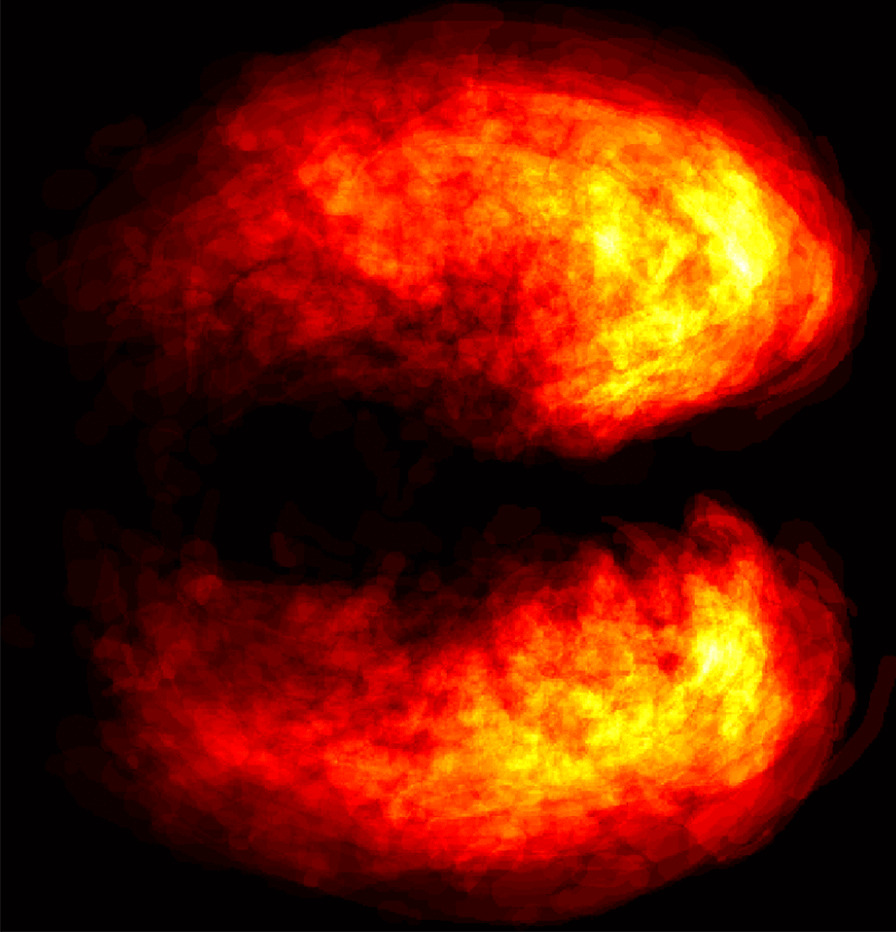
Accumulation of the dataset’s labels. All the labels of the dataset were summed up to form a graphic that illustrates the regions of the lungs most prune to infection

By visual inspection of the dataset images, we notice that the infected areas of the lungs are localized in specific regions. To illustrate the correlation between infected tissue and its relative location, all the labels of the dataset were summed and plotted with hot colormap in Fig. 2. It is clear from the accumulation image that some portions of the lungs are more prone to infection than others. Therefore, the spatial values of pixels tend to be a key feature in this research.

### Deep neural networks

**Fig. 3:**
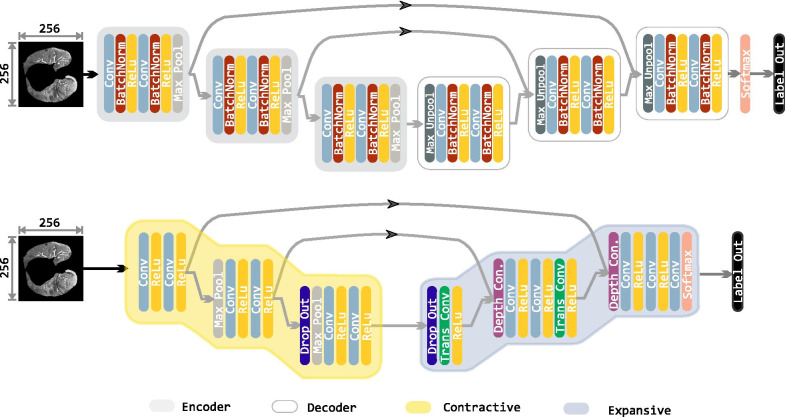
The DNN architectures The SegNet (top) where the encoder-decoder of the network are illustrated using the gray and white bubbles, and U-NET (bottom) where the contractive and expansive layer patches are encapsulated in blue and yellow bubbles

The overall methodology of semantically segmenting images is to design a structure that extracts features through successive convolutions and uses that information to create a segmentation map as an output. See Fig. 3. In the following two paragraphs, we present a brief description of the two DL networks used in this research.

#### U-NET architecture

U-NET was originally developed for medical image understanding and segmentation. It has vast applications in the domain and has been a key architecture in the medical imaging automation society. In this section, we detail the key technical features of this network and their role in achieving good results.

The architecture of this network includes two main parts: contractive and expansive. The contracting path consists of several patches of convolutions with filters of size $3\times 3$ and unity strides in both directions, followed by ReLU layers. This path extracts the key features of the input and results a feature vector of a specific length. The second path pulls information from the contractive path via copying and cropping, and from the feature vector via up-convolutions, and generates, by a successive operation, an output segmentation map. The key component of this architecture is the operation linking the first and second paths together. This linkage allows the network to attain highly accurate information from the contractive path, thus generating the segmentation mask as close as possible to the intended output. A detailed overview of the architecture can be found in [28].

#### SegNet architecture

SegNet is a Deep Neural Network originally designed to model scene segmentors such as road image segmentation tool. This task requires the network to converge using highly imbalanced datasets since large areas of road images consist of classes such as road, sidewalk, sky. In the dataset section, we demonstrated numerically how the dataset used in this work exhibit disparity in class representation. As a consequence, SegNet was our first choice for this task.

SegNet is a DNN with an encoder-decoder depth of three. The encoder layers are identical to the convolutional layers of the VGG16 network. The decoder constructs the segmentation mask by utilizing pooling indices from the max-pooling of the corresponding encoder. The creators removed the fully connected layers to reduce complexity, which reduces the number of parameters of the encoder sector from ${1.34}{{\mathrm{e}}}{+8}$ to ${1.47}{{\mathrm{e}}}{+7}$. See [29].

### Network training

Training the neural networks is done using the ADAM stochastic optimizer due to its fast convergence rate compared to other optimizers [30]. The input images are resized to $256\times 256$ to reduce the training time and also for memory requirements. The one-hundred images dataset is divided into three sets for training, validation, and testing, with proportions of 0.72, 0.10, and 0.18 respectively. In spite of class imbalance discussed earlier, class weights are calculated using median frequency balancing and handed over to the pixel classification layer to formulated a weighted cross-entropy loss function [31]:

1 $$\begin{aligned} \gamma = - \frac{1}{K} \sum _{k=1}^{K}\sum _{n=1}^{N} w_i \cdot l_k^n \cdot \mathbf{log }(p_k^n) \end{aligned}$$

where *K* is the number of instances, *N* number of classes, $l_k^n$ and $p_k^n$ are label and prediction in class *n* in instance *i*, and $w_i$ is the weight of class *i*.

**Fig. 4:**
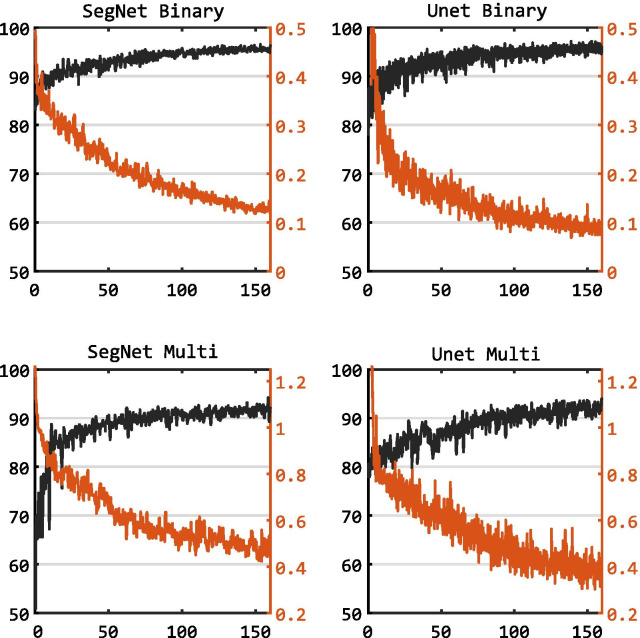
SegNet and U-NET binary and multi-class segmentors’ training accuracy and loss Four plots of training loss and accuracy for the best configuration of each segmentor

Each network is trained nine times using different hyperparameters to find the best possible configuration. Table 2 lists these training hyperparameters. For the training performance, it was completed in 160 epochs for all the experiments. Training time variation was negligible among networks, with an average of 25 min. Figure 4 illustrates training performance and loss for the best binary segmentors (U-NET #4 and SegNet #4) and the best multi-class segmentors (U-NET #4 and SegNet #7). The criteria used to conclude the best experiments are discussed in the results section.

**Table 2:** Hyperparameters used for training the DNNs

Exp.	SegNet		U-NET	
	ILR	MniBatch	ILR	MiniBatch
1	${1}{\mathrm{e}}{-4}$	4	${1}{\mathrm{e}}{-4}$	2
2	${1}{\mathrm{e}}{-4}$	8	${1}{\mathrm{e}}{-4}$	8
3	${1}{\mathrm{e}}{-4}$	12	${1}{\mathrm{e}}{-4}$	12
4	${1}{\mathrm{e}}{-3}$	4	${5}{\mathrm{e}}{-4}$	2
5	${1}{\mathrm{e}}{-3}$	8	${5}{\mathrm{e}}{-4}$	8
6	${1}{\mathrm{e}}{-3}$	12	${5}{\mathrm{e}}{-4}$	12
7	${3}{\mathrm{e}}{-3}$	4	${1}{\mathrm{e}}{-3}$	2
8	${3}{\mathrm{e}}{-3}$	8	${1}{\mathrm{e}}{-3}$	8
9	${3}{\mathrm{e}}{-3}$	12	${1}{\mathrm{e}}{-3}$	12

Nine experiments for each network with different initial learning rates (ILR) and mini batch sizes

The training process was done using the Deep Learning Toolbox version 14.0 in MATLAB R2020a (9.8.0.1323502) in a Windows 10 version 10.0.18363.959 machine with an INTEL core-i5 9400F and an NVIDIA 1050ti 4GB VRAM GPU using CUDA 10.0.130. Usage of the GPU reduced training times by a factor of 35 on average.

### Evaluation criteria and procedure

To fully quantify the performance of our models, we utilized five known classification criteria: sensitivity, specificity, G-mean, Sorensen-Dice (aka. F1), and F2 score. The following Eqs. (2)–(6) describe these criteria:

2 $$\begin{aligned} {\text {Sensitivity}} = \frac{{\text {TP}}}{{\text {TP}} + {\text {FN}}} \end{aligned}$$

3 $$\begin{aligned} {\text {Specificity}} = \frac{{\text {TN}} }{{\text {TN}} + {\text {FP}}} \end{aligned}$$

4 $$\begin{aligned} {\text {Sorensen-Dice}} = \frac{2\times {\text {TP}}}{2\times {\text {TP}} + {\text {FP}} + {\text {FN}}} \end{aligned}$$

5 $$\begin{aligned} {\text {G-mean}} = \sqrt{{\text {sensitivity}} \times {\text {specificity}}} \end{aligned}$$

6 $$\begin{aligned} {\text {F2-score}} = \frac{5\times {\text {Precision}}\times {\text {Sensitivity}}}{4\times {\text {Precision}} + {\text {Sensitivity}} } \end{aligned}$$

These criteria are selected because of the dataset imbalance nature discussed in the *Materials and Methods* section.

The evaluation was carried out as follows: the global accuracy of the classifier was calculated for each test image and averaged over all the images. Using the mean values of global accuracies, the best experiment of each network was chosen for a *”Class Level”* assessment. Then, statistical scores (2)–(6) were calculated for each class and tabulated properly.

## Results

### Binary segmentation

#### Test images results

Table  3 shows results for both models of binary classifiers after evaluating every experiment of each network. We can see from the results that our networks achieve accuracy values larger than 0.90 in all cases, and 0.954 accuracy in the best case (experiment 4 of the network SegNet). The standard deviation of experiment 4 is 0.029. The second best network is experiment 4 of the U-NET architecture with an accuracy of 0.95 and a standard deviation of 0.043.

**Table 3:**
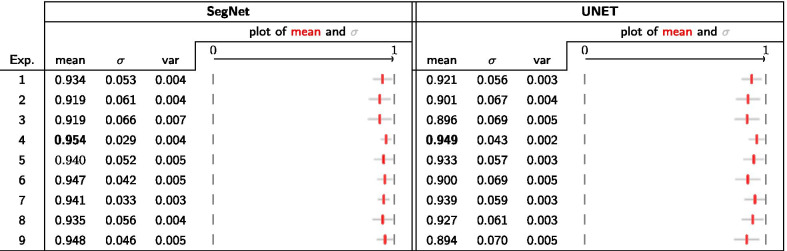
Global accuracy metrics of the Test data images calculated for the nine experiments of the UNET and SegNet networks as binary class segmentors. The “plot” columns visualize the mean accuracy and the standard deviation of each experiment

The best experiment of each architecture is selected for further performance investigation on the class level.

#### Class Level

Based on the criteria discussed in the “Methods” section, the best two networks found in the previous section are evaluated. We can see that the SegNet network surpasses U-NET with noticeable margins for all metrics except sensitivity and G-mean, where both networks produce similar results. See Table 4.

**Table 4:** Statistical results for the binary segmentor

Net.	Sens.	Spec.	Dice	G-mean	F2
SegNet	0.956	**0.9542**	**0.749**	0.955	**0.861**
U-NET	0.964	0.948	0.733	**0.956**	0.856

Bold values indicate the highest number of a comparable set

SegNet and U-NET binary segmentation tools results is terms of sensitivity, specificity, dice, G-mean, and F2 score

### Multi class segmentation

#### Test images results

Similarly, we obtain the best experiment for each multi-classification network. The best experiment of the SegNet architecture is number 7, giving an accuracy of 0.907 with a standard deviation of 0.06. We also found that the overall best accuracy of 0.908 is achieved by the fourth experiment of U-NET network with a standard deviation of 0.065. All the experiments achieve higher accuracy than 0.8 except for the first three experiments of SegNet. Refer to Table 5.

**Table 5:**
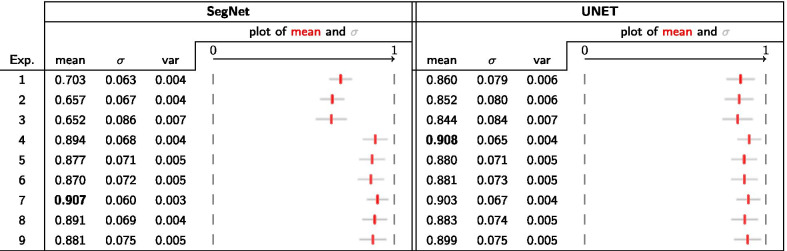
Global accuracy metrics of the Test data images calculated for the nine experiments of the UNET and SegNet networks as multi-class segmentors. The “plot” columns visualize the mean accuracy and the standard deviation of each experiment

#### Class level

In the same manner as the binary segmentation results section, the best experiment of each architecture is evaluated as presented in Table 6. Both networks struggled to recognize the C_3_ class. Nevertheless, they achieve good results for C_1_ and C_2_ . We also notice the high specificity rate regarding all the classes. The U-NET architecture recorded higher values for all parameters except the specificity.

**Table 6:** Statistical results for the multi segmentor

Net.	Class	Sens.	Spec.	Dice.	G-mean	F2
SegNet	${\hbox {C}}_1$	0.638	.**0.952**	0.479	0.780	0.562
	${\hbox {C}}_2$	0.672	0.965	0.454	0.806	0.564
	${\hbox {C}}_3$	0.574	0.988	0.121	0.753	0.231
U-NET	${\hbox {C}}_1$	**0.804**	0.930	**0.483**	**0.865**	.**0.636**
	${\hbox {C}}_2$	**0.694**	**0.983**	**0.597**	**0.826**	**0.652**
	${\hbox {C}}_3$	**0.684**	**0.993**	**0.225**	**0.824**	**0.377**

Bold values indicate the highest number of a comparable set

SegNet and U-NET multi class segmentation tools results is terms of sensitivity, specificity, dice, G-mean, and F2 score. Matching color rows display the results for the same class

## Discussion

### Binary classification problem

It can be referred from Table 5 that SegNet outperforms U-NET architecture by a noticeable margin. Both networks have an exceptionally high true positive count for the ”Not Infected” class. The results state in a quantifiable manner how reliable is the DNN models in distinguishing between the non-infected and infected classes, i.e. ill portions of the lungs. Further experiments involving a larger dataset is likely to confirm this. The high sensitivity (0.956) and specificity (0.945) of the best network (SegNet) indicate its goodness in modeling a trained radiologist for the task at hand.

Regarding the standard deviation of the results demonstrated in Table 3, the values ranged from 0.060 to 0.086. These low values indicate highly consistent accuracies in the test partition of the dataset.

The results of our SegNet show enhancements over Inf-Net and Semi-Inf-Net presented in [25] in terms of Dice, specificity, and sensitivity Metrics. As well, the U-NET outperforms them only in terms of sensitivity. Both works utilize the same dataset. As a binary segmentor, Inf-Net focuses on edge information and allocates a portion of computations to highlight it. This would remove focus from the important internal texture and allocate more weight to the fractal shaped edge, especially that no evidence of high contrast between the infection and lung tissue is found. Secondly, the parallel partial decoder used by the network gives less weights to low-level features which are considered a key for texture highlighting. Another reason might be that the SegNet was trained on dataset images that contain only lung areas.

SegNet outperforms the Semi-Inf-Net network, an architecture utilizes pseudo labeling to generate additional training data, by a small margin. This might be because the used pseudo-labeling technique generated 1600 labels from only 50 labeled images which were used to train the network.

SegNet also surpasses the COVID-SegNet architecture proposed in [22] in sensitivity and Dice metrics. This might be because, according to the authors, COVID-19 lesions were difficult to distinguish from the chest wall. COVID-SegNet was able to segment the lung region with close-to-perfect performance, yet was not able to match this accuracy in segmenting the infection regions that are close to the wall. A more detailed comparison, in which both architectures are trained and tested on the same dataset, might be necessary to further generalize this result.

It should be noted here that increasing the mini-batch size has a negative effect on the networks performance; further tests may lead to a generalized statement regarding this. A previous study investigated the mini-batch size role in the VGG16 network convergence. It concluded that smaller mini-batch sizes coupled with a low learning rate would yield a better training outcome [32]. Another study concluded that smaller mini-batch sizes tend to produce more stable training for ResNet networks by updating the gradient calculations more frequently [33].

### Multi class problem

Table 6 shows how good the U-NET is in segmenting the Ground Glass Opacification and the Consolidation. The U-NET produced moderate results in segmenting the pleural effusion; a Dice of 0.23 and F2 score of 0.38 which downplays its role as a reliable tool for pleural effusion segmentation.

The C_3_ class, as discussed in the Dataset section, is the least represented class in the dataset. Therfore, such a result is expected from a multi-class segmentation model constructed using 72 image instances only.

The standard deviation values of the multiclass segmentors were, on average, a little higher than those of the binary segmentors. Yet, they still indicate that the networks are solid performers in terms of accuracy. The high specificity rates clearly state that the models are reliable in identifying non-infected tissue (class C_0_ ).

### Five-fold cross validation

Due to the small size of images in the dataset, a five-fold cross-validation was performed as an overall assessment. The dataset images were first scrambled to form a newly randomized dataset. Then, for each iteration, images were divided into three sets: 70% for training, 10% for validation, and 20% for testing in a successive manner. The validation set was utilized for monitoring the network performance during training, and to keep the overall training data count as close as possible to the procedure performed in the *Networks Training* section.

Table 7 presents the statistical results using criteria described in the *Evaluation Criteria and Procedure* section. We notice low values of standard deviation for each score, except for the sensitivity of the ${\hbox {C}}_3$ class, with mean values close to the ones reported in Tables 4 and 6.

**Table 7:** Five-fold experiment results for the best network of each architecture

			Sensitivity		Specificity		Dice		G-mean		F2	
			$\mu$	$\sigma$	$\mu$	$\sigma$	$\mu$	$\sigma$	$\mu$	$\sigma$	$\mu$	$\sigma$
Binary	SegNet		0.947	0.048	0.945	0.015	0.703	0.055	0.945	0.019	0.829	0.029
	U-NET		0.961	0.033	0.923	0.018	0.643	0.058	0.941	0.014	0.800	0.033
Multi	SegNet	${\hbox {C}}_1$	0.653	0.043	0.927	0.030	0.425	0.084	0.778	0.035	0.535	0.069
		${\hbox {C}}_2$	0.688	0.072	0.963	0.004	0.410	0.081	0.813	0.043	0.537	0.073
		${\hbox {C}}_3$	0.679	0.270	0.987	0.006	0.117	0.068	0.804	0.167	0.214	0.110
	U-NET	${\hbox {C}}_1$	0.685	0.130	0.910	0.045	0.402	0.144	0.786	0.083	0.527	0.135
		${\hbox {C}}_2$	0.666	0.120	0.973	0.013	0.458	0.093	0.801	0.066	0.550	0.052
		${\hbox {C}}_3$	0.632	0.26	0.991	0.005	0.152	0.092	0.777	0.165	0.250	0.118

Results are the mean and standard deviation of sensitivity, specificity, dice, g-mean, and F2

### Network feature visualization

**Fig. 5:**
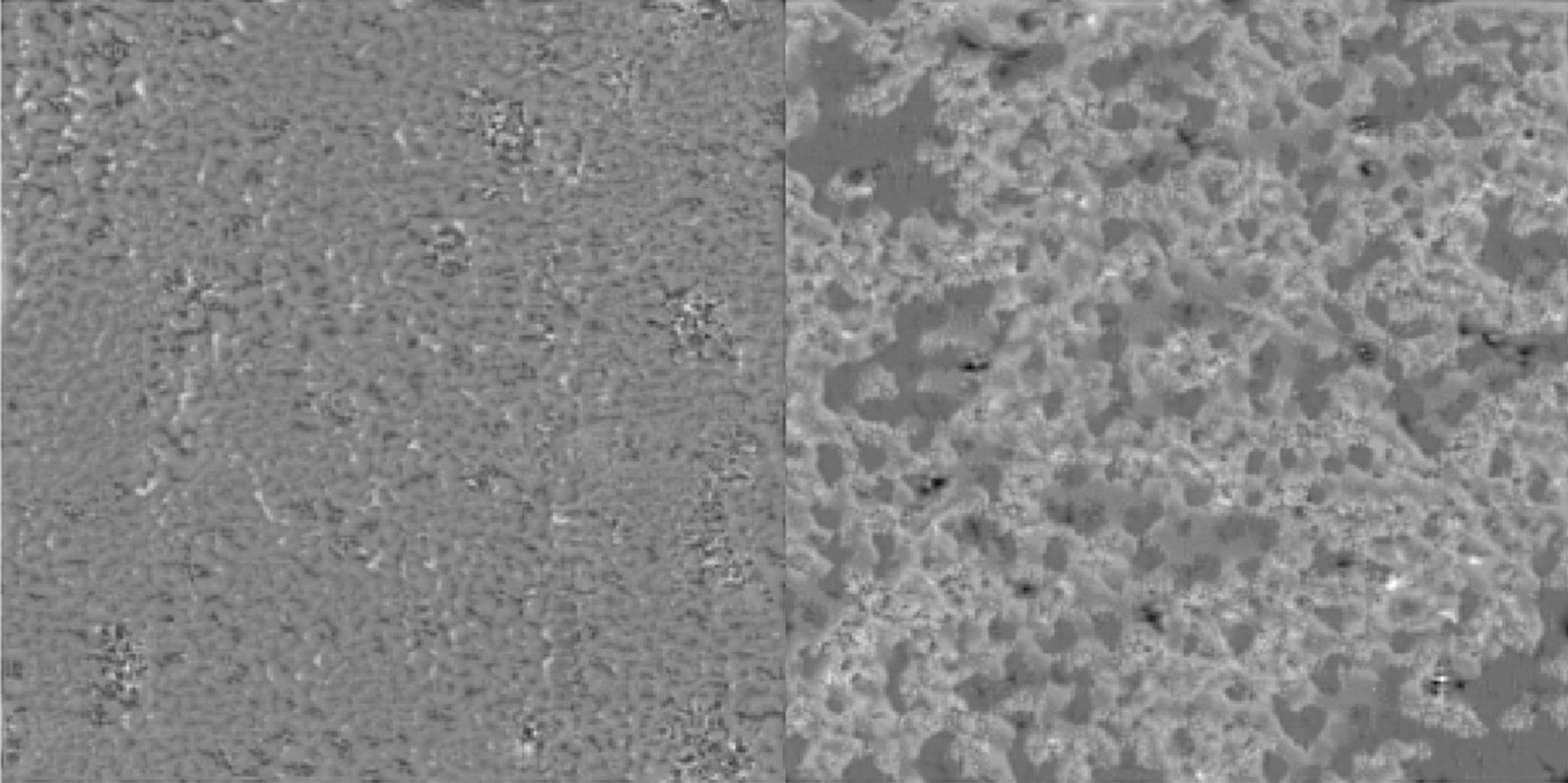
SegNet binary segmentor Deep Dream image Deep dream image laying out key features the network is using to segment the CT scans. infected tissue (right), non-infected (left)

Deep Dream is a method used to visualize the features extracted by the network after the training process [34]. Since SegNet proved to be a reliable segmentor considering its high statistical scores, the generated Deep Dream image should lay out the key features distinguishing each class (non-infected, infected). We plotted the Deep Dream image in Fig. 5. We can apparently visualize a discerning pattern between the two classes in this image.

## Conclusions

In this paper, the performance of two deep learning networks (SegNet & U-NET) was compared in their ability to detect diseased areas in medical images of the lungs of COVID-19 patients. The results demonstrated the ability of the SegNet network to distinguish between infected and healthy tissues in these images. A comparison of these two networks was also performed in a multiple classification procedure of infected areas in lung images. The results showed the U-NET network’s ability to distinguish between these areas. The results obtained in this paper represent promising prospects for the possibility of using deep learning to assist in an objective diagnosis of COVID-19 disease through CT images of the lung.

## Data Availability

The data is openly accessible in [26], and the networks used in this work are freely available in https://github.com/adnan-saood/COVID19-DL.

## Abbreviations

NIFTI: Neuroimaging informatics technology initiative
COVID-19: COrona VIrus Disease 2019
CT: Computed tomography
GGO: Ground glass opacification
DL: Deep learning
GAN: Generative adversarial networks
CNN: Convolutional neural networks
ADAM: Adaptive moment estimation
DNN: Deep neural network
